# Equine Blood and Salivary Cortisol Levels While Exercising on a Water Treadmill with an Artificial River System

**DOI:** 10.3390/ani16172631

**Published:** 2026-08-22

**Authors:** Urszula Sikorska, Dorota Lewczuk, Małgorzata Maśko

**Affiliations:** 1 Department of Animal Breeding, Institute of Animal Science, Warsaw University of Life Sciences (WULS-SGGW), Ciszewskiego 8, 02-787 Warsaw, Poland; malgorzata_masko@sggw.edu.pl; 2 Institute of Genetics and Animal Biotechnology PAN, ul. Postępu 36A, 05-551 Magdalenka, Poland; d.lewczuk@igbzpan.pl

**Keywords:** horse, stress, hydrotherapy, water counterflow, water treadmill, artificial river system

## Abstract

A water treadmill (WT) is used in equine rehabilitation and training. This study aimed to investigate whether varying water depths and using an active artificial river (AR) system during water treadmill exercise would affect physiological stress responses in leisure horses. Thirteen leisure horses participated in five 20 min WT sessions, including those with a treadmill with dry belt, fetlock-depth WT (with/without AR), and carpal-depth WT (with/without AR) at a walking speed of 1.25 m/s. Blood and salivary cortisol levels were measured before, immediately after, and 30 min after exercise. Blood cortisol levels increased after exercise in all sessions. Immediately after exercise, blood cortisol concentrations were significantly lower following the AR sessions. Salivary cortisol concentrations increased 30 min after exercise, with the highest values observed following the carpal-depth WT + AR session. The weak blood–salivary cortisol correlation likely reflects differences in temporal kinetics and biological matrices. Because all horses completed the experimental sessions in the same predefined order, the effects of AR mode cannot be completely separated from all other uncontrolled effects. However, these preliminary findings indicate that activation of the AR system modifies physiological responses during water treadmill exercise and highlight the importance of combining blood and salivary cortisol measurements when evaluating equine responses to exercise.

## Simple Summary

Water treadmills are increasingly used in horse training and rehabilitation because they allow exercise with reduced impact on the limbs while increasing muscular effort. Some water treadmills are equipped with an artificial river (AR) system that creates a water current against the horse’s movement, potentially increasing exercise intensity. However, little is known about how horses respond physiologically to this type of training. In this study, blood and salivary cortisol concentrations were measured in leisure horses exercising on a water treadmill under different depths and with the AR mode activated. Cortisol concentrations increased after exercise, confirming the activation of normal physiological responses to treadmill work. Blood cortisol levels were lower during sessions performed with the AR mode than during the corresponding non-AR sessions, whereas salivary cortisol showed a different temporal response pattern. These unique preliminary findings should be interpreted cautiously, as habituation effects cannot be completely separated from the influence of AR mode activation. The studies on blood and salivary cortisol provide complementary information and support the use of multiple physiological indicators when evaluating horses during water treadmill training.

## 1. Introduction

Domestic horses are exposed to acute and chronic stress, which may affect their health, performance, and welfare, raising significant and constant concern in modern equestrianism [1]. Stress responses involve complex psychological mechanisms, and among various stressors, physical activity is considered an important factor influencing equine physiology [1,2,3]. 

A horse’s hypothalamic–pituitary–adrenal (HPA) axis is activated in response to stress. It initiates the production of various chemicals, including glucocorticoids like cortisol [1,4]. Cortisol is widely used as a physiological indicator of HPA axis activation in response to exercise and is frequently applied in studies evaluating stress responses in horses under training conditions [5,6,7]. Cortisol has also been extensively used to evaluate physiological responses to exercise in horses, including treadmill and water treadmill training, making it a suitable indicator for assessing stress associated with different exercise modalities [7]. Aside from cortisol, endocrine responses to exercise in horses can also involve adrenaline, noradrenaline, adrenocorticotropic hormone (ACTH), insulin, growth hormone, arginine vasopressin, and thyroid hormones, whose concentrations vary according to exercise intensity, duration, and emotional context [8,9]. Nevertheless, cortisol remains one of the most widely used biomarkers because its plasma concentrations reliably reflect activation of the hypothalamic–pituitary–adrenal axis in both physiological and stress-related conditions [1,6,9]. 

Most studies have measured cortisol levels in the blood due to their reliability [4,6,10]. However, non-invasive methods have also been developed to assess cortisol levels using saliva [7,11], hair [12,13], or feces [14,15]. Non-invasive techniques are becoming increasingly popular because they avoid repeated intravenous injections, which can cause stress in horses [1]. Horses’ cortisol concentrations have been measured in various situations, such as equestrian competitions [7,16,17], training and exercise [6,7,9,18,19], transport [7,8,20], and treadmill exercises [7]. Several investigations have shown a positive correlation between cortisol levels and diverse physiological markers of stress, such as heart rate, respiratory rate, rectal temperature, eye temperature, and blood lactic acid, in horses [1,18]. 

An equine water treadmill uses water as a medium for exercise, and is utilized in equine rehabilitation and training. Treadmills and water treadmills are present in various research facilities and therapeutic centers, and are progressively gaining popularity in private competitive equestrian establishments [3]. Water treadmills serve as a hydrotherapy technique to enhance horses’ cardiovascular and muscular capabilities. Over the past few decades, the WT has emerged as a crucial instrument for various research involving horses on a global scale [7,9]. WT exercises can be individualized by adjusting factors such as water depth, belt speed, frequency of use, or exercise duration. Due to such possibilities, the effects on the horse’s condition can vary. As with any component of a horse’s training, it is crucial to align water treadmill protocols with specific training targets. 

Water-based exercise provides unique physical properties, including buoyancy and hydrodynamic resistance, which reduce joint loading while simultaneously increasing muscular effort. Studies in humans have demonstrated that aquatic resistance training improves muscle strength, functional capacity, and body composition [21]. Additionally, underwater treadmill training with water-jet resistance (artificial river, AR) has been shown to enhance gait and balance by increasing resistance and modifying locomotion patterns, highlighting the importance of controlled water flow in training systems [22]. 

The findings of Lim (2020) [22] suggest that applying the artificial river (AR) system, which generates a counter-current, may similarly increase workload and influence physiological responses in horses. 

Although cortisol has been widely used to assess physiological responses to exercise in horses, including treadmill and water treadmill studies, no studies have evaluated cortisol responses during exercise performed on a water treadmill with the artificial river (AR) mode activated. Our previous study [23], conducted on the same group of horses using the same experimental protocol, investigated cardiovascular, hematological, and biochemical responses to WT exercise, whereas the present study specifically evaluated the responses of blood and salivary cortisol levels. 

We hypothesized that incorporating AR mode would modify the physiological responses to WT exercise. Due to limited data on equine physiological responses during WT exercise with an AR, this study aimed to investigate whether varying water depths and using an active AR system during WT exercise would affect physiological stress responses in leisure horses, as assessed using blood and salivary cortisol levels.

## 2. Materials and Methods

### 2.1. Horses

Horses were selected from among the leisure riding horses at the Didactic Stable of the Horse Breeding Division at the Warsaw University of Life Sciences (WULS). The animal protocols used in this work were evaluated and approved by the II Local Ethical Committee on Animal Testing in Warsaw on behalf of the National Ethical Committees on Animal Testing (protocol code WAW2/089/2020, approved on 29 July 2020). These protocols align with FELASA guidelines and the National Law for Laboratory Animal Experimentation (Dz. U. 2015 poz. 266 and 2010-63-EU directive). 

The inclusion criteria were the absence of previous encounters with water treadmill training, no current diagnosis of orthopedic disease, no record of suboptimal performance, no clinical indicators of illness, and no indicators of lameness. Historical data regarding the animals were acquired from the training and veterinary documentation of the Didactic Stable. 

A comprehensive physical examination was performed on horses lacking a history of water treadmill exercise, orthopedic issues, or unsatisfactory performance records. The assessment involved monitoring vital parameters, including heart rate, rectal temperature, capillary refill time, respiration rate, lymph nodes, and the status of mucous membranes. Horses presenting no clinical symptoms of disease—characterized by all the measured physical parameters remaining within physiological ranges [24]—were subjected to an orthopedic examination in accordance with the standards of the American Association of Equine Practitioners (AAEP) for lameness assessment [25]. 

The present study was conducted using the same experimental protocol and the same population of horses as reported previously [23]. Thirteen leisure horses (*n* = 13) were included in this study (lameness score of 0/5) [25], comprising seven geldings and six mares, with ages ranging from 6 to 18 years, and a median age of 12. The height of the horses varied from 152 to 168 cm, with a median height of 166 cm. All the horses were Polish Warmbloods.

These horses were housed under uniform conditions at the Didactic Stable of the Horse Breeding Division at WULS. They were provided with individualized feeding regimens, factoring in their distinct nutritional requirements, which included particular proportions of hay, oats, and commercial complementary concentrate feed. Fresh water was available at all times. All the horses were leisure horses and were maintained under the same routine exercise program, consisting of approximately one hour of ridden work per day, five days per week. None of the horses were involved in competitive sport or intensive athletic training prior to the study. In addition, the horses had access to a sandy pasture for six hours each day, seven days a week. During the study period, the horses sustained their standard workload and participated in unrestricted activities within the paddock.

### 2.2. Water Treadmill Exercise

The horses were exercised on a water treadmill (Technohorse Sp. z o.o., Skarżysko-Kamienna, Poland) with an artificial river (AR) system. The AR system featured four water jets generating a constant water flow at a velocity of 3 m/s in opposition to the horse’s movement (counterflow).

Before data collection, the horses participated in a habituation protocol and behavioral assessment. The habituation protocol was taken from Greco-Otto et al. [26], while the ethogram for behavioral assessment was adapted from Maśko et al. [27]. The habituation regimen comprised three preliminary WT exercise sessions, each lasting 20 min: the initial session on a treadmill with a dry belt, the second session in fetlock-depth water, and the third session in carpal-depth water. In the last five minutes of the second and third sessions, the artificial river (AR) mode was used. The order of the sessions was not randomized. All horses followed the same predefined sequence, progressing from dry treadmill to fetlock-depth water, and then to carpal-depth water. The order of sessions was chosen for horse welfare reasons and to allow gradual familiarization with increasing exercise conditions. The order of the training exercises seemed a logical consequence of the natural conditions in the water areas. 

All sessions were conducted at a walking velocity of 1.25 m/s. Horses were classified as habituated when they performed consistent movement and did not show behavioral or heart rate-related stress indicators. The assessed stress behaviors included chewing, nostril flaring, shaking, whinnying, defecating, tail up or covered, and snorting. Furthermore, a significant increase in heart rate upon walking on the treadmill was also considered an indicator of stress. After completing the three habituation sessions, all horses were classified as habituated. 

Before starting data collection, each horse was equipped with a Polar Equine HR sensor (Polar Electro Oy, Kempele, Finland). The sensor was secured using an inflatable belt that encircled the thorax and was positioned on the left side of the horse’s chest. Heart rate data were continuously recorded during each session and subsequently summarized as mean values for each exercise session for statistical analysis. A detailed analysis of heart rate responses, together with hematological and biochemical variables collected during the same experimental protocol, has been published previously [23] and is therefore not reported here. 

The data acquisition involved five water treadmill exercise sessions, each lasting 20 min, with an additional 5 to 10 min designated for water filling and emptying depending on the water depth, during which the horse was walking at a belt speed of 1.25 m/s. All horses completed the five experimental sessions in the same predefined order. The first session was conducted on a dry treadmill (DT), followed by the second and third sessions in fetlock-depth water, and the fourth and fifth sessions in carpal-depth water. In the third and fifth sessions, the artificial river (AR) mode was activated. The sessions were classified as follows: DT, fetlock-depth WT, fetlock-depth WT + AR, carpal-depth WT, and carpal-depth WT + AR. 

The DT session served as the dry treadmill reference condition. All sessions followed the established guidelines for WT exercise in healthy horses [3]. The water depth was adjusted for each horse individually, taking limb length into account to ensure that the designated joints were adequately submerged. Each horse participated in one exercise session per week.

The environmental conditions were monitored during all the training sessions. The ambient temperature ranged from 20 to 25 °C (median: 23 °C). The humidity varied between 50% and 64% (median: 55%). The water temperature in the treadmill was consistently maintained between 13 and 15 °C (median: 14 °C). All measurements were performed at a consistent time of day (morning hours) on consecutive days in June. Three horses were evaluated per day.

### 2.3. Cortisol Level Assessment

Blood samples were obtained through jugular venipuncture using the BD Vacutainer^®^ system (Plymouth, UK) in plain tubes for serum analyses. Approximately 5 mL of blood was collected per tube. Blood samples were collected once at each time point: before exercise, immediately after, and 30 min after the treadmill session. The “before” samples were taken immediately prior to the horse entering the treadmill, while the “immediately after” samples were collected directly after the horse exited the treadmill. The collected blood samples were stored at 4 °C until centrifugation to isolate serum, within no more than 1 h of blood collection. Centrifugation was performed at 1500× *g* at 25 °C for 10 min. The serum was subsequently stored at −20 °C until assay. 

Saliva samples were collected at the same time points as blood collection. Blood samples were collected first, followed by saliva sampling. All samples were collected in the same location under consistent environmental conditions. A sponge was gently inserted into the horse’s mouth using forceps to collect saliva, wiping the sides of the cheeks, tongue, and palate for approximately 1 min. After saliva collection, the swab was inserted into a specifically labeled tube and centrifuged for 15 min at 1500× *g* to obtain saliva specimens. After centrifugation, the saliva was transferred with a pipette into 1.5 mL Eppendorf tubes and subsequently frozen at −80 °C until the assay. Blood and saliva cortisol concentrations (nmol/L) were determined using a commercially available cortisol enzyme immunoassay kit: the Cortisol ELISA kit (EA0002HO, Immuno Life, Kraków, Poland). This specific kit has been validated for the non-invasive measurement of biologically relevant changes in equine cortisol concentrations [28]. The hormone analysis protocol was established according to the manufacturer’s recommendations.

### 2.4. Statistical Analysis

Data series were tested for distribution using the Shapiro–Wilk test. The results of the normality assessment for each data series are presented in the Supplementary Materials (Tables S1 and S2). As not all the data series followed a normal distribution, medians and ranges (minimum and maximum values) are used to present data.

Two separate repeated-measures analyses were performed. The first analysis com-pared cortisol levels between sampling time points (before, immediately after, and 30 min after exercise) within each water treadmill session to evaluate the exercise effect. The second analysis compared cortisol concentrations between the water treadmill sessions (DT, fetlock-depth WT, fetlock-depth WT + AR, carpal-depth WT, and carpal-depth WT + AR) at each sampling time point to evaluate the effect of training type.

Data series were compared as paired data using repeated-measures one-way ANOVA, for normally distributed data series, or the Friedman test, for at least one data series not normally distributed. Statistical significance was set at *p* < 0.05. When significant differences were detected, Holm–Šidák’s or Dunn’s post hoc tests were used, as appropriate. Repeated-measures one-way ANOVA was followed by a post hoc Holm–Šidák multiple comparisons test. The Friedman test was followed by post hoc Dunn’s multiple comparisons test.

Given that not all the data series followed a normal distribution, Spearman’s rank correlation coefficient (ρ) was calculated to assess the correlation between the heart rate, blood cortisol level, and salivary cortisol level. Correlations were estimated for time points (before, immediately after, and 30 min after exercise) separately. Statistical significance was set at *p* < 0.05. The direction of the correlation was interpreted as positive (>0) or negative (<0). The strength of the correlations was interpreted as weak (≤0.40), moderate (0.41–0.70), strong (0.71–0.90), or very strong (≥0.91). All statistical analyses were performed using GraphPad Prism, version 6 (GraphPad Software Inc., San Diego, CA, USA).

## 3. Results

### 3.1. Exercise’s Effect on Cortisol Level (Changes Between Sampling Time Points)

The blood cortisol level significantly increased from the baseline measurement (before the treadmill session) to the subsequent measurement immediately after the treadmill session, regardless of the training type (all *p* < 0.001; Table 1). The blood cortisol levels were significantly higher at the 30 min measurement point after training compared to the measurement immediately after training in every treadmill session. 

The salivary cortisol level increased from the pre-exercise time point to 30 min after the session. No significant differences in salivary cortisol were observed between the pre-exercise measurements and those taken immediately after the sessions (all *p* < 0.001; Table 1).

### 3.2. Training Type’s Effect on Cortisol Level (Comparison Between Different Water Depths and AR Mode Activation)

This section compares cortisol concentrations among the experimental water treadmill configurations, which differed in water depth and activation of the artificial river mode.

Before exercise, the blood cortisol concentrations differed significantly between the experimental sessions (Table 2). The lowest blood cortisol concentration was observed before the carpal-depth WT + AR session, which differed significantly from the DT and fetlock-depth WT sessions. No significant differences were found among the remaining exercise conditions. 

The blood cortisol levels increased immediately after exercise in all the experimental sessions (Table 1). At this time point, the blood cortisol concentrations were significantly lower during the fetlock-depth WT + AR and carpal-depth WT + AR sessions than during the DT, fetlock-depth WT, and carpal-depth WT sessions (Table 2). Thirty minutes after exercise, no significant differences in blood cortisol levels were observed among the experimental sessions (Table 2). 

There were no differences in salivary cortisol levels between training sessions measured before and immediately after all the treadmill sessions. At 30 min after exercise, the salivary cortisol levels differed significantly between the experimental sessions (Table 2). The carpal-depth WT + AR session showed significantly higher salivary cortisol concentrations than the DT session, whereas no significant differences were observed among the fetlock-depth WT, fetlock-depth WT + AR, and carpal-depth WT sessions. 

### 3.3. Correlation Between Heart Rate and Cortisol Level

Heart rate responses during exercise sessions have been described in detail in our previous study, including comparisons between different phases of the training session [23]. In brief, HR increased in response to exercise intensity, with higher values observed in sessions involving AR mode. A positive significant correlation was observed between HR and blood cortisol level only at the time point immediately after treadmill exercises (*p* = 0.005), and it was interpreted as weak (r = 0.33). The correlations between HR and blood cortisol levels measured before and after exercise were not statistically significant. A weak positive correlation (r = 0.31) was also observed between blood cortisol and salivary cortisol levels (*p* = 0.01) at the time point measured at rest before exercise. The remaining correlations were not statistically significant (Table 3).

## 4. Discussion

The present study focused on interpreting cortisol responses in relation to different water treadmill conditions, particularly the effect of the artificial river (AR) system. In the present study, conducted in riding leisure horses, the responses of physiological factors during WT training sessions were measured. We observed the effect of WT exercise, the WT training type’s effect on blood and salivary cortisol, and the correlations between cortisol levels and HR in horses. To date, only our previous studies have investigated physiological responses during WT exercise with active AR mode in horses(Sikorska et al., 2025 [23,29]). In our first study, HR, hematological (RBC and HGB), and biochemical (CK, AST, and LDH) responses were observed during WT sessions. In our second study, surface temperature changes during water treadmill sessions, including AR mode, were assessed. It was concluded that the horse’s physiological responses to exercise on a WT with AR mode activated differed from those for other types of sessions [23]. In our second study, the surface temperature changes during water treadmill sessions, including AR mode, were measured [29]. These findings support our hypothesis that including the AR mode modifies equine physiological responses during water training. To the author’s knowledge, this is the first study in horses to monitor changes in blood and salivary cortisol levels during WT exercises with the AR system activated. To date, studies have used increasing water depths [30,31] or increasing running belt speeds [26,32,33] to intensify exercise conditions by increasing water resistance. The present study demonstrates that activating the artificial river mode can increase hydrodynamic resistance during water treadmill exercise. 

In terms of blood cortisol levels, we observed an intriguing and counterintuitive trend: lower blood cortisol levels were observed during sessions with active AR mode (both at fetlock and carpal depth) compared to during sessions without AR and sessions on a dry treadmill (DT) (Table 2). However, because all horses completed the experimental protocol in the same predefined order, these findings should be interpreted with caution, as the effects of AR mode cannot be completely separated from continued habituation or session-order effects. Because all horses fulfilled the predefined habituation criteria before the experimental phase, they were assumed to enter the study with a comparable level of adaptation to the water treadmill. Therefore, the observed decrease in blood cortisol during the AR sessions may also reflect adaptation to the repeated exercise protocol and the cumulative effects of successive training sessions rather than the independent effect of AR mode. 

This finding provides a novel perspective on cortisol responses during water treadmill exercise using an artificial river system. Previous studies have consistently reported increased cortisol levels in horses during treadmill exercise; however, these studies did not include the dynamic resistance generated by the AR system [5,9]. Importantly, Lindner et al. (2012) [33] conducted research specifically on horses exercising on standard water treadmills. It also demonstrated significant increases in physiological stress markers, including blood cortisol. Importantly, the ability of water treadmills to deliver significant physical benefits is well-documented; for instance, research demonstrates that conditioning equine athletes on water treadmills significantly improves peak oxygen consumption, a key indicator of cardiovascular fitness [34], and also leads to beneficial muscle fiber adaptations and metabolic responses in key locomotor muscles [32].

The lower blood concentrations observed during AR sessions, despite the documented increase in physical load in our previous study [23], may reflect differences in physiological responses to exercise at the systemic level that require further investigation. These compelling results suggest that different cortisol response patterns were observed during the AR sessions. However, because of the predefined session order, the underlying mechanisms remain uncertain and require confirmation in randomized studies. These findings support the concept that equine welfare assessment should integrate physiological indicators, such as cortisol levels and heart rate, together with behavioral observations rather than relying on a single parameter [7].

Furthermore, Bohák et al. (2017) [5] observed differences in cortisol responses to exercise related to individual temperament, highlighting that physiological stress responses may be influenced by multiple individual factors. Our results suggest that activating the AR mode may contribute to a better understanding of physiological responses to exercise in horses and may be useful when designing individualized training protocols. 

As expected, we observed a statistically significant increase in serum cortisol levels from the resting condition to the measurement points immediately after and 30 min after each training session on the water treadmill (Table 1). This is a typical physiological response to physical exercise [17,35], with systematic exercise testing having been shown to induce not only immediate physiological reactions but also long-term adaptive changes in horses [36]. Indeed, other studies have consistently shown that standard water treadmill sessions lead to a significant increase in both plasma and salivary cortisol levels immediately post-exercise [5,18,33]. Among those, Becker-Brick et al. (2013) [17] reported substantial cortisol release in sport horses, highlighting the physiological impact of strenuous and stressful activities. Similarly, Krieber et al. (2025) [15] observed increased fecal cortisol metabolites, indicating elevated stress levels in horses during the demanding ‘breaking-in’ period, which is the initial training phase from unridden to ridden horse. This observation is also consistent with earlier research, such as that conducted by Marc et al. (2000) [9], who demonstrated increased plasma cortisol and ACTH concentrations in Warmblood horses following standardized treadmill exercise. This confirms that exercise on the water treadmill provided a sufficient stimulus to activate the hypothalamic–pituitary–adrenal (HPA) axis. 

It is worth highlighting that, while blood cortisol levels were lower in AR sessions, the highest salivary cortisol levels at 30 min post-exercise were observed after the carpal-depth session with AR mode (Table 2). There is a significant difference between blood and saliva measurements of cortisol. Salivary cortisol is often considered a non-invasive indicator of stress, but it may be influenced by delayed secretion dynamics and reflect stress responses with a temporal lag. Salivary cortisol responses in horses are delayed compared to blood cortisol, with increases occurring approximately 20–30 min later due to diffusion processes, and peak concentrations potentially occurring even later depending on the stimulus [16,19,37]. Peeters et al. (2011) [37] showed in an ACTH challenge that, while blood cortisol concentrations peak relatively quickly, salivary cortisol responses can be delayed, reflecting different kinetic profiles. Therefore, the 30 min post-exercise sampling used in this study may not correspond to the peak salivary cortisol response, which limits direct comparison with blood cortisol data. 

Kedzierski et al. (2013) [19] demonstrated that salivary cortisol concentrations in Thoroughbred horses increased following exercise, which indicated salivary cortisol’s responsiveness to physical exertion and potential for reflecting delayed stress responses. After a physically demanding session with the AR system mode activated, there was an increase in salivary cortisol observed at 30 min post-exercise, which may reflect delayed cortisol kinetics rather than immediate stress perception. Therefore, salivary cortisol measured 30 min post-exercise may reflect the cumulative consequences of exercise rather than the immediate systemic response observed in blood. Another consideration is that the patterns of cortisol release may diverge, depending on the categorization of the stressor (physical versus psychological) and its temporal extent. The weak correlation between resting blood and salivary cortisol (Table 3) also suggests that these differences are most likely related to the time lag in cortisol secretion between blood and saliva as well as individual variability and sampling conditions, rather than reflecting fundamentally different aspects of the stress response. Unlike blood cortisol, salivary cortisol did not consistently demonstrate lower concentrations during AR sessions, supporting the view that these biological matrices reflect different temporal aspects of the physiological response and should therefore be interpreted with caution when compared directly. This temporal dissociation may partially explain the apparent discrepancy between the lower blood cortisol levels during AR sessions and higher salivary cortisol levels measured 30 min post-exercise. 

The observed weak positive correlation between heart rate (HR) and blood cortisol levels only immediately after exercise (*p* = 0.005, r = 0.33, Table 3) is consistent with the results of other studies that have also indicated a complex and not-consistently strong correlation between different stress indices in horses [2,10,19]. For example, Becker-Birck et al. (2013) [17] studied heart rate variability and cortisol release in sport horses during competitions. They revealed a complex relationship between these physiological stress indicators, which supports the concept that their correlations may strongly depend on the exercise context. This suggests that HR and cortisol may reflect different temporal dynamics of the physiological response and do not always show perfect synchrony, particularly in varying exercise situations.

A limitation of the present preliminary, novel study on the AR effect is that all the horses completed all the water treadmill sessions in the same predefined order. Consequently, the independent effects of AR mode activation, increased water depth, and repeated treadmill exposure cannot be completely separated from habituation and session-order effects. Future studies should use randomized session orders to evaluate the specific effects of AR mode activation on cortisol responses. Another limitation of the study is the relatively wide age range of the horses, which may have contributed to individual variability in cortisol responses despite the repeated-measures study design. 

## 5. Conclusions

The present study demonstrated different temporal response patterns of blood and salivary cortisol during water treadmill exercise performed under different conditions. Lower blood cortisol concentrations were observed during the AR sessions despite the greater physiological workload previously reported for the same experimental protocol [23,29]. Future randomized or counterbalanced studies are needed to determine the specific contribution of AR mode activation to physiological responses during water treadmill training. The combined assessment of blood cortisol, salivary cortisol, and heart rate provides complementary information for evaluating physiological responses to water treadmill exercise in horses.

This study highlights the importance of simultaneously monitoring multiple stress indicators, including blood and salivary cortisol, together with HR, by considering their intercorrelations. The discrepancies between blood and salivary cortisol indicate that interpreting the stress response requires a holistic approach.

## Figures and Tables

**Table 1:** The effect of exercise on blood and salivary cortisol levels measured during the following water treadmill exercise sessions: dry treadmill (DT), water treadmill (WT) session at fetlock-depth water, WT session at fetlock-depth water with artificial river (AR) mode, WT sessions at carpal-depth water, and WT session at carpal-depth water with AR mode across time points. Data are presented as median ranges (minimum and maximum values).

	Time Point	DT Session	Fetlock-Depth WT Session	Fetlock-Depth WT + AR Session	Carpal-Depth WT Session	Carpal-Depth WT + AR Session
Blood cortisol (nmol/L)	Before	89.2 (59.7–123.5) ^a^	84.4 (40.9–127.9) ^a^	69.1 (37.4–104.2) ^a^	80.6 (32.9–113.6) ^a^	45.5 (33.6–79.2) ^a^
	Immediately after	138.1 (124.4–156.7) ^b^	146.5 (130.4–158.0) ^b^	113.4 (106.5–126.0) ^b^	135.2 (115.6–149.8) ^b^	104.1 (87.5–119.9) ^b^
	30 min after	165.6 (157.0–232.0) ^c^	172.0 (158.6–204.3) ^c^	167.4 (130.0–267.1) ^c^	163.1 (153.2–206.8) ^c^	148.7 (120.0–255.3) ^c^
Salivary cortisol (nmol/L)	Before	25.5 (10.7–42.2) ^a^	28.3 (9.8–54.3) ^a^	21.2 (7.5–54.1) ^a^	16.8 (9.7–55.0) ^a^	21.7 (10.2–53.6) ^a^
	Immediately after	31.4 (11.1–57.0) ^a^	33.7 (12.2–54.7) ^a^	36.7 (10.8–49.5) ^a^	22.6 (6.7–53.6) ^a^	27.1 (8.1–55.1) ^a^
	30 min after	68.0 (60.6–126.6) ^b^	131.5 (111.9–164.8) ^b^	127.8 (94.5–190.7) ^b^	111.0 (83.2–151.6) ^b^	156.2 (116.0–183.1) ^b^
*p*-value		<0.001	<0.001	<0.001	<0.001	<0.001

Lowercase letters (a–c) indicate differences in physiological response measures across time points, supported by a *p*-value presented in the row for different sources of cortisol. Statistical significance was set at *p* < 0.05.

**Table 2:** The effect of training type on blood and salivary cortisol levels measured during the following water treadmill exercise sessions: dry treadmill (DT), water treadmill (WT) session at fetlock-depth water, WT session at fetlock-depth water with artificial river (AR) mode, WT sessions at carpal-depth water, and WT session at carpal-depth water with AR mode. Data are presented as median ranges (minimum and maximum values).

	Time Point	DT Session	Fetlock-Depth WT Session	Fetlock-Depth WT + AR Session	Carpal-Depth WT Session	Carpal-Depth WT + AR Session	*p*-Value
Blood cortisol (nmol/L)	Before	89.2 (59.7–123.5) ^a^	84.4 (40.9–127.9) ^a^	69.1 (37.4–104.2) ^ab^	80.6 (32.9–113.6) ^ab^	45.5 (33.6–79.2) ^b^	=0.005
	Immediately after	138.1 (124.4–156.7) ^a^	146.5 (130.4–158.0) ^a^	113.4 (106.5–126.0) ^b^	135.2 (115.6–149.8) ^a^	104.1 (87.5–119.9) ^b^	<0.001
	30 min after	165.6 (157.0–232.0) ^a^	172.0 (158.6–204.3) ^a^	167.4 (130.0–267.1) ^a^	163.1 (153.2–206.8) ^a^	148.7 (120.0–255.3) ^a^	=0.13
Salivary cortisol (nmol/L)	Before	25.5 (10.7–42.2) ^a^	28.3 (9.8–54.3) ^a^	21.2 (7.5–54.1) ^a^	16.8 (9.7–55.0) ^a^	21.7 (10.2–53.6) ^a^	=0.21
	Immediately after	31.4 (11.1–57.0) ^a^	33.7 (12.2–54.7) ^a^	36.7 (10.8–49.5) ^a^	22.6 (6.7–53.6) ^a^	27.1 (8.1–55.1) ^a^	=0.51
	30 min after	68.0 (60.6–126.6) ^a^	131.5 (111.9–164.8) ^b^	127.8 (94.5–190.7) ^b^	111.0 (83.2–151.6) ^b^	156.2 (116.0–183.1) ^c^	<0.001

Lowercase letters (a–c) indicate differences in physiological response measures across DT/WT exercise sessions, supported by a *p*-value presented in the column. Statistical significance was set at *p* < 0.05.

**Table 3:** Correlations between heart rate (HR), blood cortisol, and salivary cortisol levels during water treadmill exercise sessions at the following measured time points: before, immediately after, and 30 min after. Data are presented as Spearman’s rank correlations (r_s_). Statistical significance was set at *p* < 0.05.

Correlations	Time Point	r_s_	*p*-Value
HR Blood cortisol level	Before	0.13	0.29
	Immediately after	0.33	0.005
	30 min after	−0.06	0.62
HR Salivary cortisol level	Before	0.22	0.08
	Immediately after	−0.14	0.26
	30 min after	−0.24	0.05
Blood cortisol level Salivary cortisol level	Before	0.31	0.01
	Immediately after	0.12	0.32
	30 min after	−0.13	0.29

The direction of the correlation was interpreted as positive (>0) or negative (<0). The strength of the correlations was interpreted as weak (≤0.40), moderate (0.41–0.70), strong (0.71–0.90), or very strong (≥0.91).

## Data Availability

The raw data supporting the conclusions of this article will be made available by the authors on request.

## Supplementary Materials

The following supporting information can be downloaded at: https://www.mdpi.com/article/10.3390/ani16172631/s1, Table S1: Results of the Shapiro–Wilk normality test for the exercise-effect analysis; Table S2: Results of the Shapiro–Wilk normality test for the training-type analysis.

## References

[1] Sikorska U., Maśko M., Ciesielska A., Zdrojkowski Ł., Domino M. (2023). Role of Cortisol in Horse’s Welfare and Health. Agriculture.

[2] Pawluski J., Jego P., Henry S., Bruchet A., Palme R., Coste C., Hausberger M. (2017). Low Plasma Cortisol and Fecal Cortisol Metabolite Measures as Indicators of Compromised Welfare in Domestic Horses (Equus Caballus). PLoS ONE.

[3] Nankervis K., Tranquille C., McCrae P., York J., Lashley M., Baumann M., King M., Sykes E., Lambourn J., Miskimmin K.A. (2021). Consensus for the General Use of Equine Water Treadmills for Healthy Horses. Animals.

[4] Kirchmeier A., van Herwaarden A.E., van der Kolk J.H., Sauer F.J., Gerber V. (2020). Plasma Steroid Profiles before and after ACTH Stimulation Test in Healthy Horses. Domest. Anim. Endocrinol..

[5] Bohák Z., Szenci O., Harnos A., Kutasi O., Kovács L. (2017). Effect of Temperament on Cortisol Response to a Single Exercise Bout in Thoroughbred Racehorses—Short Communication. Acta Vet. Hung..

[6] Cravana C., Medica P., Fazio E., Satué K., Brancato G., La Fauci D., Bruschetta G. (2025). Circulating ACTH and Cortisol Investigations in Standardbred Racehorses Under Training and Racing Sessions. Vet. Sci..

[7] Massányi M., Halo M., Mlyneková E., Kováčiková E., Tokárová K., Greń A., Massányi P. (2023). The Effect of Training Load Stress on Salivary Cortisol Concentrations, Health Parameters and Hematological Parameters in Horses. Heliyon.

[8] Ferlazzo A., Fazio E., Medica P. (2020). Behavioral Features and Effects of Transport Procedures on Endocrine Variables of Horses. J. Vet. Behav..

[9] Marc M., Parvizi N., Ellendorff F., Kallweit E., Elsaesser F. (2000). Plasma Cortisol and ACTH Concentrations in the Warmblood Horse in Response to a Standardized Treadmill Exercise Test as Physiological Markers for Evaluation of Training Status. J. Anim. Sci..

[10] Hovey M.R., Davis A., Chen S., Godwin P., Porr C.A.S. (2021). Evaluating Stress in Riding Horses: Part One—Behavior Assessment and Serum Cortisol. J. Equine Vet. Sci..

[11] Keating D.L., Lehman J.S., Burk S.V. (2021). Salivary Cortisol, Equine Characteristics, and Management Factors Associated With Strongyle-Type Egg Shedding of Ohio Horses. J. Equine Vet. Sci..

[12] Sauveroche M., Henriksson J., Theodorsson E., Svensson Holm A.-C., Roth L.S.V. (2020). Hair Cortisol in Horses (*Equus caballus*) in Relation to Management Regimes, Personality, and Breed. J. Vet. Behav..

[13] Saluti G., Ricci M., Castellani F., Colagrande M.N., Di Bari G., Vulpiani M.P., Cerasoli F., Savini G., Scortichini G., D’Alterio N. (2022). Determination of Hair Cortisol in Horses: Comparison of Immunoassay vs LC-HRMS/MS. Anal. Bioanal. Chem..

[14] Christensen J.W., Strøm C.G., Nicová K., de Gaillard C.L., Sandøe P., Skovgård H. (2022). Insect-Repelling Behaviour in Horses in Relation to Insect Prevalence and Access to Shelters. Appl. Anim. Behav. Sci..

[15] Krieber J., Nowak A.C., Geissberger J., Illichmann O., Macho-Maschler S., Palme R., Dengler F. (2025). Fecal Cortisol Metabolites Indicate Increased Stress Levels in Horses During Breaking-In: A Pilot Study. Animals.

[16] Peeters M., Closson C., Beckers J.F., Vandenheede M. (2013). Rider and Horse Salivary Cortisol Levels During Competition and Impact on Performance. J. Equine Vet. Sci..

[17] Becker-Birck M., Schmidt A., Lasarzik J., Aurich J., Möstl E., Aurich C. (2013). Cortisol Release and Heart Rate Variability in Sport Horses Participating in Equestrian Competitions. J. Vet. Behav..

[18] Casella S., Vazzana I., Giudice E., Fazio F., Piccione G. (2016). Relationship between Serum Cortisol Levels and Some Physiological Parameters Following Reining Training Session in Horse. Anim. Sci. J..

[19] Kedzierski W., Strzelec K., Cywińska A., Kowalik S. (2013). Salivary Cortisol Concentration in Exercised Thoroughbred Horses. J. Equine Vet. Sci..

[20] Lertratanachai S., Poochipakorn C., Sanigavatee K., Huangsaksri O., Wonghanchao T., Charoenchanikran P., Lawsirirat C., Chanda M. (2024). Cortisol Levels, Heart Rate, and Autonomic Responses in Horses during Repeated Road Transport with Differently Conditioned Trucks in a Tropical Environment. PLoS ONE.

[21] Martínez-Rodríguez A., Cuestas-Calero B.J., Martínez-Olcina M., Marcos-Pardo P.J. (2021). Benefits of Adding an Aquatic Resistance Interval Training to a Nutritional Education on Body Composition, Body Image Perception and Adherence to the Mediterranean Diet in Older Women. Nutrients.

[22] Lim C.G. (2020). Effect of Underwater Treadmill Gait Training With Water-Jet Resistance on Balance and Gait Ability in Patients With Chronic Stroke: A Randomized Controlled Pilot Trial. Front. Neurol..

[23] Sikorska U., Maśko M., Rey B., Domino M. (2025). Heart Rate, Hematological, and Biochemical Responses to Exercise on Water Treadmill with Artificial River in School Horses. Appl. Sci..

[24] Costa L.R.R. (2017). History and Physical Examination of the Horse. Man. Clin. Proced. Horse.

[25] Keegan K.G., Dent E.V., Wilson D.A., Janicek J., Kramer J., Lacarrubba A., Walsh D.M., Cassells M.W., Esther T.M., Schiltz P. (2010). Repeatability of Subjective Evaluation of Lameness in Horses. Equine Vet. J..

[26] Greco-Otto P., Bond S., Sides R., Kwong G.P.S., Bayly W., Léguillette R. (2017). Workload of Horses on a Water Treadmill: Effect of Speed and Water Height on Oxygen Consumption and Cardiorespiratory Parameters. BMC Vet. Res..

[27] Masko M., Domino M., Lewczuk D., Jasinski T., Gajewski Z. (2020). Horse Behavior, Physiology and Emotions during Habituation to a Treadmill. Animals.

[28] Share E.R., Mastellar S.L., Suagee-Bedore J.K., Eastridge M.L. (2024). Validation of a Commercial ELISA Kit for Non-Invasive Measurement of Biologically Relevant Changes in Equine Cortisol Concentrations. Animals.

[29] Sikorska U., Maśko M., Rey B., Domino M. (2025). Utility of Infrared Thermography for Monitoring of Surface Temperature Changes During Horses’ Work on Water Treadmill with an Artificial River System. Animals.

[30] McCrae P., Bradley M., Rolian C., Léguillette R. (2021). Water Height Modifies Forelimb Kinematics of Horses during Water Treadmill Exercise. Comp. Exerc. Physiol..

[31] Tranquille C., Tacey J., Walker V., Mackechnie-Guire R., Ellis J., Nankervis K., Newton R., Murray R. (2022). Effect of Water Depth on Limb and Back Kinematics in Horses Walking on a Water Treadmill. J. Equine Vet. Sci..

[32] Firshman A.M., Borgia L.A., Valberg S.J. (2015). Effects of Training at a Walk on Conventional and Underwater Treadmills on Fiber Properties and Metabolic Responses of Superficial Digital Flexor and Gluteal Muscles to High-Speed Exercise in Horses. Am. J. Vet. Res..

[33] Lindner A., Wäschle S., Sasse H.H.L. (2012). Physiological and Blood Biochemical Variables in Horses Exercising on a Treadmill Submerged in Water. J. Anim. Physiol. Anim. Nutr..

[34] Greco-Otto P., Bond S., Sides R., Bayly W., Leguillette R. (2020). Conditioning Equine Athletes on Water Treadmills Significantly Improves Peak Oxygen Consumption. Vet. Rec..

[35] Malinowski K., Shock E.J., Rochelle P., Kearns C.F., Guirnalda P.D., Mckeever K.H. (2006). Plasma Beta-Endorphin, Cortisol and Immune Responses to Acute Exercise Are Altered by Age and Exercise Training in Horses. Equine Vet. J. Suppl..

[36] Čebulj-Kadunc N., Frangež R., Kruljc P. (2025). Long-Term Changes of Physiological Reactions in Young Lipizzan Stallions During Exercise Testing. Animals.

[37] Peeters M., Sulon J., Beckers J.F., Ledoux D., Vandenheede M. (2011). Comparison between Blood Serum and Salivary Cortisol Concentrations in Horses Using an Adrenocorticotropic Hormone Challenge. Equine Vet. J..

