# Supplementary material for: Equine Blood and Salivary Cortisol Levels While Exercising on a Water Treadmill with an Artificial River System
Source: Animals (Basel). 2026 Aug 22;16(17):2631. doi: 10.3390/ani16172631 (PMC13564286; doi:10.3390/ani16172631)
Supplement: Supplementary file 1 [file animals-16-02631-s001.zip › animals-4414275-supplementary.pdf]

Supplementary Data for

Equine Blood and Salivary Cortisol Levels While Exercising on a Water Treadmill with an Artificial River System

Urszula Sikorska [1*], Dorota Lewczuk [2] and Małgorzata Maśko [1,]

[1] Department of Animal Breeding, Institute of Animal Science, Warsaw University of Life Sciences (WULS-SGGW), Ciszewskiego 8, 02-787 Warsaw, Poland; malgorzata_masko@sggw.edu.pl

[2] Institute of Genetics and Animal Biotechnology PAN, ul. Postępu 36A, 05-551 Magdalenka, Poland; d.lewczuk@igbzpan.pl

* Correspondence: urszula_sikorska@sggw.edu.pl; Tel.: +48-508-286-768

Table S1: Results of the Shapiro–Wilk normality test for the exercise-effect analysis during the following water treadmill exercise sessions: dry treadmill (DT), water treadmill (WT) session at fetlock-depth water, WT session at fetlock-depth water with artificial river (AR) mode, WT sessions at carpal-depth water, and WT session at carpal-depth water with AR mode.

Variable	Time point	DT	Fetlock-Depth WT	Fetlock-Depth WT + AR	Carpal-Depth WT	Carpal-Depth WT + AR
Blood cortisol	Before	Yes	Yes	Yes	Yes	Yes
	Immediately after	Yes	Yes	Yes	Yes	Yes
	30 min after	No	No	Yes	No	No
Salivary cortisol	Before	Yes	Yes	Yes	No	Yes
	Immediately after	Yes	Yes	No	Yes	Yes
	30 min after	No	Yes	Yes	Yes	Yes

Yes indicates that the data followed a normal distribution according to the Shapiro–Wilk test ($p > 0.05$), whereas **No** indicates that the data did not follow a normal distribution ($p \leq 0.05$).

Table S2: Results of Shapiro-Wilk normality test for the training-type analysis during the following water treadmill exercise sessions: dry treadmill (DT), water treadmill (WT) session at fetlock-depth water, WT session at fetlock-depth water with artificial river (AR) mode, WT sessions at carpal-depth water, and WT session at carpal-depth water with AR mode.

Variable	Time point	DT	Fetlock-Depth WT	Fetlock-Depth WT + AR	Carpal-Depth WT	Carpal-Depth WT + AR
Blood cortisol	Before	Yes	Yes	Yes	Yes	Yes
Blood cortisol	Immediately after	Yes	Yes	Yes	Yes	Yes
Blood cortisol	30 min after	No	No	Yes	No	No
Salivary cortisol	Before	Yes	Yes	Yes	No	Yes

Salivary cortisol	Immediately after	Yes	Yes	No	Yes	Yes
Salivary cortisol	30 min after	No	Yes	Yes	Yes	Yes

Yes indicates that the data followed a normal distribution according to the Shapiro–Wilk test ($p > 0.05$), whereas **No** indicates that the data did not follow a normal distribution ($p \leq 0.05$).
